# Beyond Rotavirus: Persistence of Norovirus, Adenovirus, and Astrovirus in Paediatric Gastroenteritis in the Republic of Congo After Vaccine Introduction

**DOI:** 10.1002/jmv.70896

**Published:** 2026-04-13

**Authors:** Cedeche Lebraiche Durain Mboungou, Claujens Chastel Mfoutou Mapanguy, Do Duc Anh, Jeannhey Christevy Vouvoungui, Alain Maxime Mouanga, Vivaldie Mikounou Louya, Emmanuel Seun Kupoluyi, Raoul Ampa, Thirumalaisamy P. Velavan, Francine Ntoumi

**Affiliations:** ^1^ Fondation Congolaise pour la Recherche Médicale, Brazzaville Republic of Congo; ^2^ Faculty of Sciences and Techniques, Marien Ngouabi University Brazzaville Republic of Congo; ^3^ Institute of Tropical Medicine, University of Tübingen and German Center for Infection Research (DZIF) Tübingen Germany; ^4^ Vietnamese—German Center for Medical Research (VG‐CARE) Hanoi Vietnam; ^5^ Faculty of Health Sciences, Marien Ngouabi University Brazzaville Republic of Congo; ^6^ Faculty of Medicine Duy Tan University Da Nang Vietnam

**Keywords:** adenovirus, astrovirus, gastroenteritis, norovirus, Republic of Congo, rotavirus

## Abstract

Despite the introduction of rotavirus vaccines, gastroenteritis remains a major cause of paediatric morbidity and mortality in the Republic of Congo. This study investigated the prevalence and genetic diversity of non‐rotavirus enteric viruses including norovirus (NoV), adenovirus (AdV), and astrovirus (AstV) ten years after the introduction of the rotavirus vaccine in 2014. A cross‐sectional study was conducted between 2022 and 2023 among 227 children (≤ 5 years old) hospitalised for acute gastroenteritis in Brazzaville. Stool samples were analysed by multiplex RT‐PCR to detect RVA, NoV, AdV, and AstV. Positive AdV and NoV samples underwent genotyping by PCR and sequencing. The incidence of gastroenteritis peaked during the dry season, with the highest burden observed among children aged 6‐24 months. Overall, 75% (*n* = 170/227) of children tested positive for at least one virus; RVA was detected in 58% (*n* = 131/227), followed by NoV in 34% (*n* = 77/227), AdV in 11% (*n* = 24/227), and AstV in 7% (*n* = 16/227), while 25% (*n* = 57/227) tested negative for all four viruses. Co‐infections were observed in 32% of children (*n* = 72/227), most commonly RVA‐NoV. The predominant genotype of norovirus was GII.P31 (82%), and that of adenovirus was type 41 (67%). Notably, from November 2022, gastroenteritis of unknown aetiology peaked, coinciding with a decline in the four targeted viruses. NoV, AstV, and AdV continue to contribute to the gastroenteritis burden in Congolese children. The high circulation of NoV and the seasonal surge of unexplained cases highlight the need for broadened molecular surveillance, incorporation of bacterial diagnostics, and consideration of future NoV vaccine strategies.

## 1 Introduction

Diarrhoeal diseases remain a leading cause of morbidity and mortality in children worldwide [1]. These illnesses cause an estimated 2.5 billion cases and approximately 1.6–2.5 million deaths annually in children under five, with more than 70% of fatal cases occurring in Africa and Southeast Asia [2]. Despite advances in prevention and treatment, diarrhoeal diseases continue to represent a major public health challenge in low‐ and middle‐income countries. A substantial proportion of cases present as acute gastroenteritis, characterised by diarrhoea, vomiting, and dehydration, which may progress to severe complications or death if not appropriately managed [3].

The aetiology of gastroenteritis involves a wide range of pathogens, including bacteria and parasites, with enteric viruses remaining the major contributors [3]. Across sub‐Saharan Africa, rotavirus A (RVA) has consistently been the leading cause of acute gastroenteritis in infants and young children over the past decade, followed by norovirus (NoV) [4, 5]. Other viruses, such as sapovirus, astrovirus (AstV), and adenovirus (AdV; types 40/41), have also been implicated in paediatric diarrhoeal disease in the region [6, 7]. Their contribution is often under‐recognised because these infections present with clinical features that overlap with other causes of diarrhoea, and limited access to advanced diagnostic testing further hampers accurate case identification [3].

The WHO officially recommended the implementation of rotavirus vaccines across all national immunisation programmes in 2009 [8]. This strategy has shown promising outcomes in reducing the prevalence of rotavirus in several countries [9]. In the Republic of Congo, the rotavirus vaccine Rotarix™ was introduced in the national immunisation programme in April 2014 [10]. In 2020, Rotarix™ was subsequently replaced by Rotavac®, which is also a monovalent, live attenuated oral rotavirus vaccine. Our previous study showed that the introduction of rotavirus vaccination was associated with an approximately 20% decline in RVA prevalence among hospitalised children under 5 years of age, with vaccine coverage reaching 43% [11]. However, other studies in the region have reported that although RVA prevalence declined following vaccine introduction, unusual genotypes continued to circulate, and other enteric viruses such as NoV, AstV, and AdV gained greater relative importance in childhood diarrhoea [5, 12]. For instance, rotavirus surveillance in Eastern and Southern Africa showed the circulation of multiple genotypes both before and after vaccine rollout, including low‐frequency atypical/unusual G/P combinations such as G1P[4], G2P[8], G9P[4] and G12P[4] [13]. In addition, post‐rotavirus vaccine surveillance in Kenya revealed a significant decline in the prevalence of rotavirus from 23.3% to 13.8%; conversely, the prevalence of norovirus increased from 6.6% to 10.9%, while adenovirus prevalence rose from 5% to 7% [14, 15]. However, data on the genetic diversity of these viruses remain limited in Congo, particularly following the introduction of the rotavirus vaccine in 2014.

In this context, our present study investigates the prevalence, genetic diversity, and molecular epidemiology of NoV, AstV, and AdV in hospitalised children with acute gastroenteritis in Brazzaville, Republic of Congo.

## 2 Material and Methods

### 2.1 Study Location and Ethical Approval

This cross‐sectional observational study was carried out in two healthcare centres located in southern Brazzaville, Republic of Congo: The Referral Hospital of Makélékélé (HRM) and the Basic Hospital of Bacongo (HBB). Stool samples collected at both sites were transported within 24 h to the Centre de Recherche sur les Maladies Infectieuses Christophe Mérieux (CeRMI‐CM) of the Fondation Congolaise pour la Recherche Médicale (FCRM) for analysis. The study was reviewed and approved by the Institutional Ethics Committee of the Congolese Foundation for Medical Research (Approval number: 035/CEI/FCRM/2021).

### 2.2 Patient Recruitment and Sample Collection

Patient recruitment was conducted over a 12‐month period, from April 2022 to March 2023. Children were enrolled by the attending clinician following clinical examination, after written informed consent was obtained from a parent or legal guardian. Eligibility criteria comprised age ≤ 5 years and hospitalisation for gastroenteritis, defined as the passage of three or more loose stools within a 24 h period (Bristol stool scale 5–7), with or without accompanying abdominal pain, dehydration, vomiting, or fever (≥ 37°C). For each participant, a stool sample was collected within 48 h of admission. Samples were transported within 24 h to CeRMI‐CM and stored at −20°C until analysis.

### 2.3 Nucleic Acid Extractions and cDNA Synthesis

Prior to processing, stool samples were diluted 10‐fold in phosphate‐buffered saline (PBS, 1X). The suspensions were centrifuged at 14,000 rpm (rotation per minute) for 3 min, and 140 μL of the supernatant was used for nucleic acid extraction. RNA was extracted using the QIAamp Viral RNA Mini Kit and DNA was extracted using the QIAamp DNA Mini Kit (Qiagen, Hilden, Germany), according to the manufacturer's instructions. Nucleic acids were eluted in 60 μL of elution buffer and stored at −80°C until analysis.

Extracted RNA from each sample was reverse‐transcribed using the LunaScript™ RT SuperMix Kit (New England Biolabs, Ipswich, MA, USA). Reverse transcription was performed with 8 µL of RNA and 2 µL LunaScript RT SuperMix (5X), under the following conditions: 25°C for 2 min, 55°C for 10 min, and 95°C for 1 min. The transcription was carried out on a SimpliAmp thermal cycler (Applied Biosystems, Thermo Fisher Scientific, Singapore).

### 2.4 Detection of Adenovirus, Norovirus and Astrovirus

The RVA infection rate was assessed and reported in our previous study using the same study cohort [11]. For adenoviruses, AdV types 40 and 41 were detected by conventional PCR using genotype‐specific primers (Supplementary Table 1), as previously described [16, 17]. In brief, a volume of 18 µL of master mix was prepared containing 2.5 µL of 10X Buffer, 0.5 µL of 10 mM dNTPs, 0.5 µL of 10 µM for each primer and 0.2 µL of DNA Taq polymerase enzyme, the remaining volume was completed with RNase free water. In each corresponding tube a 2 µL volume of extracted DNA (approx. 100 ng) was added. Amplification was carried out on a SimpliAmp thermal cycler (Applied Biosystems, Thermo Fisher Scientific, Singapore) under the following conditions: an initial denaturation at 94°C for 2 min, followed by 40 cycles of denaturation at 94°C for 1 min, annealing at 62°C for 1 min, and extension at 72°C for 1 min. A final extension was performed at 72°C for 2 min. PCR products were electrophoresed on a 1.2% agarose gel and visualised under UV illumination.

Detection of NoV and AstV infections was performed by PCR using specific primers (Supplementary Table 1), as previously described [18, 19]. In brief, 8.3 µL of master mix was prepared, consisting of 2.5 µL of 10X buffer, 0.5 µL of 10 mM dNTPs, 0.6 µL of 10 µM of each primer, 0.5 µL of Taq DNA polymerase, 0.2 µL of RNasin, and RNase‐free water to the final volume. To each tube, 2 µL of synthesised cDNA (~100 ng) was added to the appropriately labelled reaction tube. Amplification was carried out on a SimpliAmp thermal cycler (Applied Biosystems, Thermo Fisher Scientific, Singapore) under the following conditions: reverse transcription at 50°C for 30 min, initial denaturation at 94°C for 15 min, followed by 40 cycles of denaturation at 94°C for 30 s, annealing at 42°C for 30 s, and extension at 72°C for 30 s. A final extension was performed at 72°C for 90 s. PCR products were electrophoresed on a 1.2% agarose gel and visualised under UV illumination.

### 2.5 NoV Genotyping by Sequencing

NoV genotyping was conducted through capsid gene amplification to differentiate between genogroups I and II [20]. Subsequently, NoV sub‐genotyping was carried out using Oxford Nanopore sequencing (ONT) in NoV‐positive samples. Among the 56 NoV‐positive samples, 25 were selected for ONT sequencing based on sample and reagent availability. Amplicons generated from NoV capsid gene amplification were purified using a magnetic bead method, and the eluted DNA was quantified with Qubit (DNA BR assay, Thermo Scientific, Singapore). DNA from each sample was then normalised to 100 ng in 7.5 µL, followed by the addition of 2.5 µL of the corresponding barcode using the SQK‐RBK110.96 kit (Oxford Nanopore Technologies, Oxford, UK).

After incubation (30°C, 1 min; 80°C, 1 min; 4°C, 1 min) in a thermocycler, up to 50 barcoded samples were pooled into a single tube and purified using Ampure XP beads (Beckman Coulter, Brea, CA, USA). The pooled eluate (11 µL) constituted the sequencing library, to which 1 µL of RAP (Rapid Adapter, Oxford Nanopore Technologies) was immediately added. For sequencing, a final mixture was prepared by combining 37.5 µL of sequencing buffer and 25.5 µL of loading beads with the library and then loaded onto an ONT GridION flow cell according to the Rapid Barcoding protocol (SQK‐RBK110.96).

### 2.6 Bioinformatics Analysis

The FastQ files generated from sequencing were first demultiplexed into individual files corresponding to each barcode used during library preparation. Each file then underwent the following steps: (i) quality control assessment, (ii) filtering of low‐quality reads, (iii) mapping against reference viral gene sequences retrieved from GenBank, (iv) alignment visualisation using Integrative Genomics Viewer (IGV v2.16.2) [21] to assess depth and coverage, and (v) generation of a final FASTA consensus sequence. These steps were integrated into an in‐house pipeline incorporating Nanoplot (v1.42.0) [22], Nanofilt (v2.8.0) [23], Minimap2 (v 2.28‐r1209) [24], Samtools (v1.20) [25] and Ivar (v1.4.3) [26].

Based on the sequencing and bioinformatics success criteria, we obtained 17 samples that passed all the filters. Sequences with > 80% coverage were submitted individually to GenBank BLAST (nucleotide) to identify the closest genotype match. In parallel, all sequences were analysed using the Genome Detective Virus Tool (v2.94) [27] for cross‐validation. The sequences generated from this study were submitted to GenBank and were assigned the accession numbers (GenBank IDs: OR225882 to OR225898).

### 2.7 Statistical Analysis

Data were analysed and visualised using R version 4.3.2 (http://www.r-project.org). Demographic and clinical data were presented as median values (with ranges) for quantitative variables and absolute numbers (with percentages) for categorical variables. The normality of the distribution in the quantitative variables was tested using the Shapiro‐Wilk test. Categorical data were compared using the Chi‐square or Fisher's exact test, and continuous variables using Student's t‐test or the Wilcoxon test, as appropriate. Tests were defined as statistically significant if the *p*‐value was < 0.05.

## 3 Results

### 3.1 Patients' Characteristics on Admission

A total of 227 paediatric patients diagnosed with acute gastroenteritis were included in the study, comprising 59% (*n* = 134/227) males and 41% (*n* = 93/227) females. The median age was 11 months (range: 1–58 months), and the median duration of hospitalisation was 5 days (range: 1–8 days). Overall, 43% (n = 98/227) of children had received at least one dose of either Rotavac® or Rotarix™ vaccine (Table 1).

**Table 1:** Demographic and clinical characteristics of patients at admission.

	Study population (*n* = 227)	Missing data *n* (%)
Sex (male)	134 (59%)	0 (0%)
Age (months)	11 [1–58]	0 (0%)
Weight (kg)	7 [2–16]	19 (8%)
Hospitalisation length (days)	5 [1–8]	0 (0%)
Vaccinated at least 1 dose (Rotavac®/Rotarix^TM^)/Un‐Vaccinated	98 (43%)/63 (27%)	66 (29%)
Clinical Presentation		
Fever	170 (75%)	4 (2%)
Vomiting	169 (74%)	0 (0%)
Vomiting episodes	3 [0–10]	4 (2%)
Vomiting duration (days)	2 [0–7]	35 (15%)
Abdominal pain	71 (31%)	2 (1%)
Nausea	2 (1%)	4 (2%)
Headaches	2 (1%)	4 (2%)
Convulsions	2 (1%)	3 (1%)
Diarrheal	227 (100%)	0 (0%)
Diarrheal episodes/day	3 [1–10]	5 (2%)
Stool consistency (Bristol Stool Form Scale)	Watery: 154 (68%), Mushy: 42 (19%), Soft blobs: 28 (12%)	3 (1%)
Dehydration level	Light: 110 (49%), Moderate: 100 (44%), Severe: 6 (3%)	11 (5%)

*Note:* Data were presented as median [with ranges] for quantitative variables and absolute numbers with percentages (%) for categorical variables.

The majority of children presented with typical clinical manifestations of gastroenteritis, with diarrhoea reported in 100% of cases (*n* = 227), fever in 75% (*n* = 170/227), and vomiting in 74% (*n* = 169/227). Abdominal pain was reported in 31% (*n* = 71/227), whereas nausea and headache were uncommon, each occurring in two children. Convulsions were also observed in two children (Table 1). Based on the Bristol Stool form scale, stool was predominantly watery (68%, type 7), followed by mushy stool (19%, type 6) and soft blobs with clear cut edges (12%, type 5). Dehydration was observed in 95% (*n* = 216/227) of children and was classified as mild in 49% (*n* = 110/227), moderate in 44% (*n* = 100/227), and severe in 3% (*n* = 6/227) (Table 1).

### 3.2 Distribution of Enteric Viruses in the Study Population

**Figure 1:**
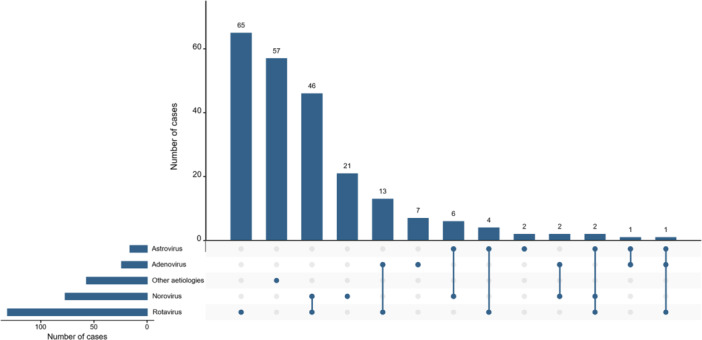
Distribution of enteric viruses among hospitalised children with acute gastroenteritis.

Among 227 hospitalised children with acute gastroenteritis, 75% (*n* = 170/227) tested positive for at least one of the four targeted enteric viruses, while 25% (*n* = 57/227) remained of unknown aetiology. RVA was the most frequently detected virus, identified in 58% of children (*n* = 131/227), as reported in our previous study [11], followed by NoV in 34% (*n* = 77/227), AdV in 11% (*n* = 24/227), and AstV in 7% (*n* = 16/227). Co‐infections were observed in 32% (*n* = 72/227) of children, with the RVA‐NoV combination being the most common (Figure 1).

**Figure 2:**
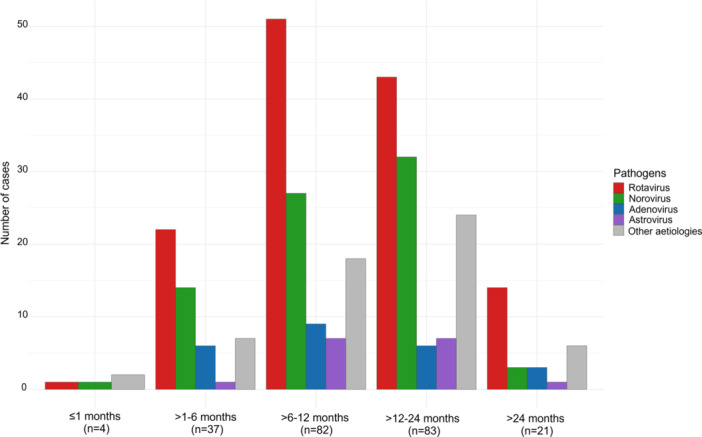
Age‐specific prevalence of enteric viruses among hospitalised children.

The highest detection rate of viral gastroenteritis was observed in children aged 6−24 months, with a marked decline in prevalence among children below 6 months and over 24 months (Figure 2). Across all age groups, RVA incidence peaked in the 6−12 months‐old group, whereas NoV and unknown aetiology were most observed in the 12−24 months‐old group, highlighting an age‐related shift in the distribution of gastroenteritis aetiologies.

**Figure 3:**
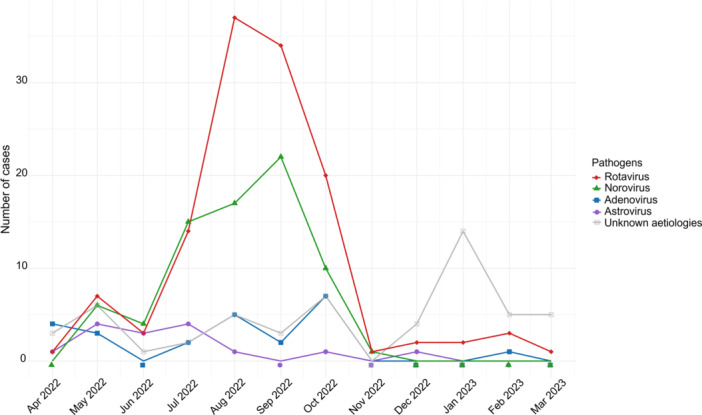
Monthly distribution of enteric virus infections during the study period.

A seasonal pattern was observed in Figure 3, where total viral gastroenteritis cases, particularly RVA and NoV, peaked during the dry season (June‐October), with the highest burden in August‐September. In contrast, AdV and AstV were detected throughout the year without a clear seasonal trend. Notably, beginning in November 2022, there was a surge in gastroenteritis cases caused by unknown aetiology, which coincided with a decline in detection of all four targeted viruses (Figure 3).

### 3.3 Genotype Distribution of Norovirus and Adenovirus

Genotyping revealed limited diversity among circulating NoV and AdV strains (Table 2). Of the 17 NoV‐positive samples successfully genotyped, the majority (82%) belonged to GII.P31, while GI.P5 (12%) and GI.P3 (6%) were detected at lower frequencies. Among the 24 genotyped AdV cases, AdV41 predominated (67%), followed by AdV40 (25%), and a small proportion of patients (8%) showing co‐infection with both AdV40 and AdV41 (Table 2).

**Table 2:** Genotype distribution of norovirus and adenovirus detected in hospitalised children.

	Norovirus (*n* = 17)			Adenovirus (*n* = 24)		
	GI.P3	GI.P5	GII.P31	AdV40	AdV41	AdV40&41
Number of cases (%)	1 (6%)	2 (12%)	14 (82%)	6 (25%)	16 (67%)	2 (8%)

*Note:* GI and GII: Norovirus genogroups I and II, respectively; *P* indicates the polymerase genotype. AdV40 and AdV41 correspond to adenovirus types 40 and 41.

### 3.4 Comparison of Clinical Presentations in Vaccinated and Unvaccinated Children

Abdominal pain was more frequent in unvaccinated children than in vaccinated children (49% vs 31%, *p* = 0.03) (Table 3). However, vaccinated children experienced more episodes of vomiting per day (median episodes: four vs. three, *p* = 0.02) compared to unvaccinated children, and liquid stools were also more frequent among vaccinated children (81% vs. 56%, *p* < 0.001). No significant differences were observed in the presence of fever, hospitalisation length, or dehydration levels.

**Table 3:** Comparison of clinical characteristics between vaccinated and unvaccinated children.

	Vaccinated (*n* = 98)	Unvaccinated (*n* = 63)	*p* value
Age (months)	9 [2–29]	11 [0–36]	0.95
Sex (male)	67 (68.4%)	28 (44.4%)	0.04
Hospitalisation length (days)	4 [2–8]	5 [1–8]	0.99
Weight (kg)	7.30 [4.10–12.0]	7.00 [2.00–15.0]	0.09
Presence of fever	76 (77.6%)	47 (74.6%)	0.72
Temperature (°C)	38.0 [36.0–40.0]	38.0 [36.7–40.0]	0.14
Vomiting	74 (75.5%)	48 (76.2%)	1
Number of vomit (episodes)	4 [1–9]	3 [1–7]	0.02
Duration of vomiting (days)	2 [1–7]	2 [1–8]	0.76
Presence of diarrheal	97 (99.0%)	63 (100%)	1
Number of diarrheal (episodes)	6 [1–13]	5 [2–13]	0.08
Bristol Stool Form Scale			
Watery (type 7)	79 (80.6%)	35 (55.6%)	< 0.001
Mushy (type 6)	8 (8.2%)	19 (30.2%)	
Soft blobs (type 5)	10 (10.2%)	9 (14.3%)	
Missing	1 (1.0%)	0 (0%)	
Dehydration level			
Light	42 (42.9%)	30 (47.6%)	0.78
Moderate	50 (51.0%)	29 (46.0%)	
Severe	1 (1.0%)	1 (1.6%)	
Missing	5 (5.1%)	3 (4.8%)	
Abdominal pain	30 (30.6%)	31 (49.2%)	0.03
Convulsion	1 (1.0%)	1 (1.6%)	1
Enteric virus positivity			
Rotavirus positive	59 (60%)	34 (54%)	0.54
Norovirus positive	34 (35%)	22 (35%)	1
Adenovirus positive	9 (9%)	7 (11%)	0.90
Astrovirus positive	6 (6%)	9 (14%)	0.14
Unknown aetiology	24 (24%)	15 (24%)	1

*Note:* Data were presented as median [with ranges] for quantitative variables and absolute numbers (with percentages) for categorical variables. Categorical data were compared using the chi‐square or Fisher's exact test, as appropriate, and continuous variables using Student's *t*‐test or the Wilcoxon test. Tests were defined as statistically significant if the *p*‐value was < 0.05.

With respect to the distribution of gastroenteritis pathogens, in the vaccinated group (*n* = 98), RVA was detected in 60%, NoV in 35%, AdV in 9%, and AstV in 6% of cases. In the unvaccinated group (*n* = 63), detection rates were 54%, 35%, 11%, and 14%, respectively. No significant differences were observed in the distribution of enteric viruses between the two groups (Table 3).

## 4 Discussion

Enteric viruses remain a leading cause of childhood gastroenteritis. This study updates the molecular epidemiology of NoV, AdV, and AstV among Congolese children hospitalised with acute gastroenteritis, nearly a decade after the introduction of the rotavirus vaccine. Our study reveals that besides RVA, other viruses such as NoV, AdV, and AstV make substantial contributions to the disease incidence.

Before the introduction of the RVA vaccine in the Republic of Congo, NoV was detected in 27% of hospitalised paediatric diarrhoea cases [28]. In our study, NoV was found in roughly one‐third of cases, indicating its persistently high circulation among Congolese children. NoV‐RVA co‐infections were frequently observed, similar to earlier reports from 2012 to 2013 [4]. This reinforces the point that NoV has established itself as a major aetiological agent of paediatric diarrhoea, often ranking just behind RVA [29].

AstV and AdV were also observed as important contributors in our study cohort. AstV was detected in approximately 10% of hospitalised children with diarrhoea, aligning with prior reports from Congo and Gabon during 2012−2013 (the pre‐vaccine era) [6, 7]. Although AstV generally causes milder illness than RVA or NoV, its consistent detection highlights a long‐term existence among young children and supports its recognition as a common viral aetiology in Africa [28, 29]. Similarly, AdV has been reported as the third most important viral cause of paediatric diarrhoea [30], and its particular year‐round circulation observed in our study underscores the epidemiological importance. The continued and consistent detection of AstV and AdV emphasizes that these viruses have remained present both before and after years of RVA vaccine application, underlining their sustained role in the burden of paediatric gastroenteritis.

Our analysis revealed relatively limited genotype diversity of enteric viruses circulating in the Republic of Congo during 2022−2023. For NoV, infections were mostly caused by genogroup GII viruses, while only a small proportion of cases were associated with genogroup GI. The predominance of the NoV GII genotype in our study (82%) was comparable to that observed in the pre‐vaccine era (87%) [28]. Notably, the GII.P31 genotype has been frequently reported to be associated with the globally prevalent GII.4 Sydney 2012 capsid lineage, which has been the principal cause of acute gastroenteritis worldwide for more than a decade [31, 32]. Given the genetic diversity of NoV, it is essential to monitor the local evolution of NoV to better understand its epidemiology and impact in sub‐Saharan Africa [29].

AdV genotyping revealed AdV41 as the most prevalent strain, followed by AdV40 and a few mixed AdV40‐AdV41 infections. This distribution reflects the well‐recognised predominance of AdV41 over AdV40 in paediatric diarrhoea, consistent with patterns previously reported in Congo and other countries in the pre‐vaccine era [30, 33]. A multicentre hospital‐based study in Kano, Nigeria, during the same period detected only AdV41 among children under five with diarrhoea, further confirming its dominance in sub‐Saharan Africa [34]. AdV41 has been associated not only with higher prevalence but also with more severe clinical manifestations compared to AdV40 in gastrointestinal disease, highlighting its particular importance [35]. Although enteric adenoviruses 40 and 41 predominate, non‐enteric adenovirus types, such as AdV31 (species A), have also been reported in paediatric diarrhoea cases; however, the contribution of non‐enteric AdV types was not assessed in this study [36].

With respect to annual incidence, our study reveals a distinct age‐related pattern of paediatric gastroenteritis in Congo. Viral infections peaked in children aged 6‐24 months and declined sharply in both younger and older age groups, reflecting the heightened vulnerability of this age range. Infants under 6 months may benefit from protection through breast milk, while children older than 24 months are more likely to have acquired immunity from past infections. In Brazzaville's 2012−2013 surveillance, NoV also occurred only in children under 24 months, peaking at 7−12 months [28]. A similar distribution was observed in our study, with RVA and NoV clustering among infants aged 6−24 months. Studies from Nicaragua and Gabon also report NoV and AstV predominance in the first 2 years of life [7, 37]. Collectively, these findings highlight the consistent association between specific pathogens and age in paediatric diarrhoeal diseases. Moreover, hospitalisations due to RVA and NoV peaked during the dry season (June‐October), with the highest incidence observed between August and September. This pattern mirrors earlier reports from Brazzaville [28], and global evidence showing RVA and NoV peaks in drier, cooler tropical seasons. By contrast, AdV and AstV circulated year‐round without clear seasonality, consistent with their reported endemic transmission across diverse climatic conditions [38]. However, the hospital‐based and urban nature of the study population in southern Brazzaville may limit the generalisability of the findings to the entire population of the region, including those of rural settings, private healthcare users, and children with limited access to formal healthcare services.

A notable observation in our surveillance was the surge in gastroenteritis cases of unknown aetiology beginning in November 2022, coinciding with a decline in the four viral pathogens tested. This emergence suggests a possible outbreak or increased circulation of other diarrhoeal pathogens not captured by our assays. These may include untested viral agents such as sapovirus, as well as bacterial pathogens including *Escherichia coli*, *Shigella spp*., or *Vibrio cholerae*. *Shigella*, in particular, remains a leading cause of severe childhood diarrhoeal disease in Africa and is second only to rotavirus in terms of mortality burden [38]. Particularly, many unexplained cases occurred in children aged 12‐24 months, a group particularly vulnerable to *Shigella* and other bacterial pathogens. While the Republic of Congo did not officially report a major cholera outbreak in late 2022, sporadic cases in the region underscore the plausibility of bacterial epidemics [39]. These observations highlight the urgent need to incorporate bacterial and additional viral pathogens beyond the common enteric viruses into surveillance efforts to guide more accurate diagnosis and enable better‐targeted treatment and prevention strategies.

In addition, comparison between vaccinated and unvaccinated children yielded varied results. Among children who had received at least one dose of the RVA vaccine, RVA was still detected in 60% of cases compared with 54% in unvaccinated children, suggesting incomplete protection or strain‐specific immunity [9, 12]. Our previously published data revealed substantial genetic diversity of RVA circulating during 2022–2023, with P[8] as the predominant P genotype, followed by P[4], P[6], and P[9], and G3, G4, and G1 as the most frequently detected G types [11]. Common strain combinations included G3P[8], G2P[4], and G3P[9], indicating the co‐circulation of both homotypic and heterotypic viruses. This genetic diversity includes strains that differ from the monovalent vaccine strains used in the region, namely Rotarix™ (G1P[8]) and Rotavac® (G9P[11]). The detection of RVA infections in vaccinated children is therefore more likely to reflect partial heterotypic protection and non‐sterilizing immunity rather than true vaccine failure, particularly in high‐transmission settings.

Regarding the clinical manifestations, although abdominal pain was more frequently reported in unvaccinated children, vaccinated children presented with higher rates of vomiting, while no significant differences were observed between the groups for fever, dehydration, or duration of hospitalisation. These counterintuitive patterns should be interpreted with caution, given the relatively small sample size and the presence of incomplete vaccination schedules. Further large‐scale longitudinal studies are warranted to clarify these associations and to provide a deeper understanding of the RVA vaccine's impact on gastroenteritis and other diarrhoeal illnesses.

## 5 Conclusion

Nearly a decade after rotavirus vaccine introduction, norovirus, astrovirus, and adenovirus continue to account for a substantial proportion of paediatric diarrhoeal diseases in Brazzaville, mirroring patterns observed during the pre‐vaccine era. Norovirus remains the leading non‐rotavirus pathogen, with persistent dominance of GII strains, while astrovirus and adenovirus type 41 show sustained circulation. These findings underscore the need for expanded surveillance and prevention strategies that extend beyond rotavirus alone.

## Author Contributions

Francine Ntoumi and Thirumalaisamy P. Velavan conceptualized, supervised the study, and contributed to the study materials and assays. Claujens Chastel Mfoutou Mapanguy, Cedeche Lebraiche Durain Mboungou, Emmanuel Seun Kupoluyi, and Vivaldie Mikounou Louya recruited the patients and contributed to the investigation materials for sampling procedures. Claujens Chastel Mfoutou Mapanguy, Cedeche Lebraiche Durain Mboungou, and Vivaldie Mikounou Louya performed the experimental procedures. Do Duc Anh, Claujens Chastel Mfoutou Mapanguy, and Jeannhey Christevy Vouvoungui performed the data curation, formal analysis, validation, and visualization of the results. Do Duc Anh, Claujens Chastel Mfoutou Mapanguy, and Cedeche Lebraiche Durain Mboungou wrote the first draft. Thirumalaisamy P. Velavan, Francine Ntoumi, Do Duc Anh, Claujens Chastel Mfoutou Mapanguy, Raoul Ampa, and Alain Maxime Mouanga reviewed the first draft. All authors have read and approved the manuscript.

## Ethics Statement

Children were enrolled by the attending clinician following clinical examination, after written informed consent was obtained from a parent or legal guardian. The study was reviewed and approved by the Institutional Ethics Committee of the Congolese Foundation for Medical Research (Approval number: 035/CEI/FCRM/2021).

## Conflicts of Interest

The authors declare no conflicts of interest.

## Supporting information


**Supplementary Table 1:** Primer list for enteric viruses PCR.

## Data Availability

The data that support the findings of this study are available from the corresponding author upon reasonable request. The authors confirm that the data supporting the findings of this study are available within the article and are also available on request.

## References

[1] H. H. Kyu , A. Vongpradith , R.‐M. V. Dominguez , et al., “Global, Regional, and National Age‐Sex‐Specific Burden of Diarrhoeal Diseases, Their Risk Factors, and Aetiologies, 1990–2021, for 204 Countries and Territories: A Systematic Analysis for the Global Burden of Disease Study 2021,” Lancet Infectious Diseases 25, no. 5 (2025): 519–536. 39708822 10.1016/S1473-3099(24)00691-1 PMC12018300

[2] K. S. Kouame , M. E. Verga , A. Pittet , et al., “[Zinc and Diarrhea in Children Under 5 Years: Who Recommendations Implemented in Switzerland],” Revue medicale suisse 8, no. 344 (2012): 1244–1247. 22730622

[3] T. G. Flynn , M. P. Olortegui , and M. N. Kosek , “Viral Gastroenteritis,” Lancet 403, no. 10429 (2024): 862–876. 38340741 10.1016/S0140-6736(23)02037-8

[4] V. Mikounou Louya , B. Nguekeng Tsague , F. Ntoumi , C. Vouvoungui , and S. C. Kobawila , “High Prevalence of Norovirus and Rotavirus Co‐Infection in Children With Acute Gastroenteritis Hospitalised in Brazzaville, Republic of Congo,” Tropical Medicine & International Health 24, no. 12 (2019): 1427–1433. 31627250 10.1111/tmi.13317

[5] C. Troeger , I. A. Khalil , P. C. Rao , et al., “Rotavirus Vaccination and the Global Burden of Rotavirus Diarrhea Among Children Younger Than 5 Years,” JAMA Pediatrics 172, no. 10 (2018): 958–965. 30105384 10.1001/jamapediatrics.2018.1960 PMC6233802

[6] B. Nguekeng Tsague , V. Mikounou Louya , F. Ntoumi , et al., “Occurrence of Human Astrovirus Associated With Gastroenteritis Among Congolese Children in Brazzaville, Republic of Congo,” International Journal of Infectious Diseases 95 (2020): 142–147. 32194237 10.1016/j.ijid.2020.02.056

[7] G. P. Manouana , P. A. Nguema‐Moure , M. Mbong Ngwese , et al., “Genetic Diversity of Enteric Viruses in Children under Five Years Old in Gabon,” Viruses 13, no. 4 (2021): 545. 33805214 10.3390/v13040545 PMC8064335

[8] P.‐I. Lee , P.‐Y. Chen , Y.‐C. Huang , et al., “Recommendations for Rotavirus Vaccine,” Pediatrics and neonatology 54, no. 6 (2013): 355–359. 23746943 10.1016/j.pedneo.2013.03.019

[9] B. D. Hallowell , J. Tate , and U. Parashar , “An Overview of Rotavirus Vaccination Programs in Developing Countries,” Expert Review of Vaccines 19, no. 6 (2020): 529–537. 32543239 10.1080/14760584.2020.1775079 PMC9234970

[10] L. H. Lenguiya , F. R. Niama , P. I. Mayengue , et al., “Molecular Study of Rotavirus A Infection in Children With Diarrhea, Before and After Vaccine Introduction in Brazzaville and Pointe‐Noire, Republic of the Congo,” Archives of Microbiology & Immunology 7, no. 1 (2023): 8.

[11] C. L. D. Mboungou , C. C. Mfoutou Mapanguy , A. M. Mouanga , et al., “The Prevalence of Rotavirus Infection Among Congolese Children Younger Than 5 Years Hospitalized for Gastroenteritis 10 Years After Introduction of Rotavirus Vaccination,” IJID Regions 14 (2025): 100596. 40144537 10.1016/j.ijregi.2025.100596 PMC11938071

[12] J. M. Mwenda , U. D. Parashar , A. L. Cohen , and J. E. Tate , “Impact of Rotavirus Vaccines in Sub‐Saharan African Countries,” Vaccine 36, no. 47 (2018): 7119–7123. 29914848 10.1016/j.vaccine.2018.06.026 PMC11726318

[13] L. M. Seheri , N. B. Magagula , I. Peenze , et al., “Rotavirus Strain Diversity in Eastern and Southern African Countries Before and After Vaccine Introduction,” Vaccine 36, no. 47 (2018): 7222–7230. 29203181 10.1016/j.vaccine.2017.11.068

[14] A. W. Lambisia , S. Onchaga , N. Murunga , C. S. Lewa , S. G. Nyanjom , and C. N. Agoti , “Epidemiological Trends of Five Common Diarrhea‐Associated Enteric Viruses Pre‐ and Post‐Rotavirus Vaccine Introduction in Coastal Kenya,” Pathogens 9, no. 8 (2020): 660. 32824245 10.3390/pathogens9080660 PMC7459961

[15] C. N. Agoti , M. D. Curran , N. Murunga , et al., “Differences in Epidemiology of Enteropathogens in Children Pre‐ and Post‐Rotavirus Vaccine Introduction in Kilifi, Coastal Kenya,” Gut Pathogens 14, no. 1 (2022): 32. 35915480 10.1186/s13099-022-00506-z PMC9340678

[16] S. Jiang , H. Dezfulian , and W. Chu , “Real‐Time Quantitative Pcr for Enteric Adenovirus Serotype 40 in Environmental Waters,” Canadian Journal of Microbiology 51, no. 5 (2005): 393–398. 16088334 10.1139/w05-016

[17] N. Jothikumar , T. L. Cromeans , V. R. Hill , X. Lu , M. D. Sobsey , and D. D. Erdman , “Quantitative Real‐Time Pcr Assays for Detection of Human Adenoviruses and Identification of Serotypes 40 and 41,” Applied and Environmental Microbiology 71, no. 6 (2005): 3131–3136. 15933012 10.1128/AEM.71.6.3131-3136.2005 PMC1151802

[18] D. Y. Oh , G. Gaedicke , and E. Schreier , “Viral Agents of Acute Gastroenteritis in German Children: Prevalence and Molecular Diversity,” Journal of Medical Virology 71, no. 1 (2003): 82–93. 12858413 10.1002/jmv.10449

[19] S. R. Finkbeiner , L. R. Holtz , Y. Jiang , et al., “Human Stool Contains a Previously Unrecognized Diversity of Novel Astroviruses,” Virology Journal 6 (2009): 161. 19814825 10.1186/1743-422X-6-161 PMC2765957

[20] S. Kojima , T. Kageyama , S. Fukushi , et al., “Genogroup‐Specific Pcr Primers for Detection of Norwalk‐Like Viruses,” Journal of Virological Methods 100, no. 1–2 (2002): 107–114. 11742657 10.1016/s0166-0934(01)00404-9

[21] H. Thorvaldsdottir , J. T. Robinson , and J. P. Mesirov , “Integrative Genomics Viewer (Igv): High‐Performance Genomics Data Visualization and Exploration,” Briefings in Bioinformatics 14, no. 2 (2013): 178–192. 22517427 10.1093/bib/bbs017 PMC3603213

[22] W. De Coster and R. Rademakers , “NanoPack2: Population‐Scale Evaluation of Long‐Read Sequencing Data,” Bioinformatics 39, no. 5 (2023): btad311, https://pubmed.ncbi.nlm.nih.gov/37171891/. 37171891 10.1093/bioinformatics/btad311 PMC10196664

[23] W. De Coster , S. D'Hert , D. T. Schultz , M. Cruts , and C. Van Broeckhoven , “Nanopack: Visualizing and Processing Long‐Read Sequencing Data,” Bioinformatics 34, no. 15 (2018): 2666–2669. 29547981 10.1093/bioinformatics/bty149 PMC6061794

[24] H. Li , “Minimap2: Pairwise Alignment for Nucleotide Sequences,” Bioinformatics 34, no. 18 (2018): 3094–3100. 29750242 10.1093/bioinformatics/bty191 PMC6137996

[25] P. Danecek , J. K. Bonfield , J. Liddle , et al., “Twelve Years of SAMtools and Bcftools,” GigaScience 10, no. 2 (2021): giab008, https://pubmed.ncbi.nlm.nih.gov/33590861/. 33590861 10.1093/gigascience/giab008 PMC7931819

[26] N. D. Grubaugh , K. Gangavarapu , J. Quick , et al., “An Amplicon‐Based Sequencing Framework for Accurately Measuring Intrahost Virus Diversity Using PrimalSeq and iVar,” Genome Biology 20, no. 1 (2019): 8. 30621750 10.1186/s13059-018-1618-7 PMC6325816

[27] M. Vilsker , Y. Moosa , S. Nooij , et al., “Genome Detective: An Automated System for Virus Identification From High‐Throughput Sequencing Data,” Bioinformatics 35, no. 5 (2019): 871–873. 30124794 10.1093/bioinformatics/bty695 PMC6524403

[28] V. Mikounou Louya , C. Vouvoungui , F. Koukouikila‐Koussounda , F. Veas , S. C. Kobawila , and F. Ntoumi , “Molecular Characterization of Norovirus Infection Responsible for Acute Diarrhea in Congolese Hospitalized Children under Five Years Old in Brazzaville, Republic of Congo,” International Journal of Infectious Diseases 88 (2019): 41–48. 31382046 10.1016/j.ijid.2019.07.034

[29] B. A. Lopman , D. Steele , C. D. Kirkwood , and U. D. Parashar , “The Vast and Varied Global Burden of Norovirus: Prospects for Prevention and Control,” PLoS Medicine 13, no. 4 (2016): e1001999. 27115709 10.1371/journal.pmed.1001999 PMC4846155

[30] P. Khales , M. H. Razizadeh , S. Ghorbani , et al., “Human Adenoviruses in Children With Gastroenteritis: A Systematic Review and Meta‐Analysis,” BMC Infectious Diseases 24, no. 1 (2024): 478. 38724898 10.1186/s12879-024-09386-x PMC11084101

[31] H. Ushijima , S. A. Hoque , Y. Akari , et al., “Molecular Evolution of GII.P31/GII.4_Sydney_2012 Norovirus over a Decade in a Clinic in Japan,” International Journal of Molecular Sciences 25, no. 7 (2024): 3619. 38612429 10.3390/ijms25073619 PMC11011564

[32] N. Navarro‐Lleó , C. Santiso‐Bellón , S. Vila‐Vicent , et al., “Recombinant Noroviruses Circulating in Spain From 2016 to 2020 and Proposal of Two Novel Genotypes Within Genogroup I,” Microbiology Spectrum 10, no. 4 (2022): e02505‐21. 35862999 10.1128/spectrum.02505-21 PMC9430863

[33] G. Mayindou , B. Ngokana , A. Sidibé , et al., “Molecular Epidemiology and Surveillance of Circulating Rotavirus and Adenovirus in Congolese Children With Gastroenteritis,” Journal of Medical Virology 88, no. 4 (2016): 596–605. 26378607 10.1002/jmv.24382

[34] M. B. Umar , A. O. Akande , L. D. Rogo , et al., “Enteric Adenovirus Gastroenteritis in Under‐Five Children, Kano‐Nigeria,” Nigerian Journal of Basic and Clinical Sciences 21, no. 3 (2024): 207–211.

[35] A. A. Rabaan , M. A. Bakhrebah , M. S. Nassar , et al., “Suspected Adenovirus Causing an Emerging Hepatitis Among Children Below 10 Years: A Review,” Pathogens 11, no. 7 (2022): 712. 35889958 10.3390/pathogens11070712 PMC9317240

[36] T. Lion , “Adenovirus Persistence, Reactivation, and Clinical Management,” FEBS Letters 593, no. 24 (2019): 3571–3582. 31411731 10.1002/1873-3468.13576

[37] S. Becker‐Dreps , F. Bucardo , S. Vilchez , et al., “Etiology of Childhood Diarrhea After Rotavirus Vaccine Introduction: A Prospective, Population‐Based Study in Nicaragua,” Pediatric Infectious Disease Journal 33, no. 11 (2014): 1156–1163. 24879131 10.1097/INF.0000000000000427 PMC4216626

[38] L. G. do Nascimento , A. M. Fialho , J. S. R. de Andrade , R. M. S. de Assis , and T. M. Fumian , “Human Enteric Adenovirus F40/41 as a Major Cause of Acute Gastroenteritis in Children in Brazil, 2018 to 2020,” Scientific Reports 12, no. 1 (2022): 11220. 35780169 10.1038/s41598-022-15413-1 PMC9250496

[39] WHO ., “ Cholera ‐ Democratic Republic of the Congo 2023, https://www.who.int/emergencies/disease-outbreak-news/item/2023-DON441#:~:text=Cholera%20is%20endemic%20in%20parts,26%20provinces%20of%20the%20country.

