# Supplementary material for: Beyond Rotavirus: Persistence of Norovirus, Adenovirus, and Astrovirus in Paediatric Gastroenteritis in the Republic of Congo After Vaccine Introduction
Source: J Med Virol. 2026 Apr 13;98(4):e70896. doi: 10.1002/jmv.70896 (PMC13071788; doi:10.1002/jmv.70896)
Supplement: Supplementary file 1 — Supplementary Table 1: Primer list for enteric viruses PCR. [file JMV-98-e70896-s001.docx]

**Supplementary Table 1: Primer list for enteric viruses PCR**

|  | **Primer sequences** | **Pathogens** | **Genes** | **Amplicon size** |
| --- | --- | --- | --- | --- |
| 1 | SF0073-F: GATTGGACTCGATTTGATGG | Human Astrovirus  (AstV) | Polymerase ARN | 400 - 500 bp |
| 2 | SF0076-R: CTGGCTTAACCCACATTCC |  |  |  |
| 3 | Ad40-F: ACCCACGATGTAACCACAGACA | Human Adenovirus 40  (AdV-40) | Polymerase | 88 - 120 bp |
| 4 | Ad40-R: ACTTTGTAAGAGTAGGCGGTTTCC |  |  |  |
| 5 | Ad41--F: TGGCCACCCCCTCGATGA | Human Adenovirus 41  (AdV-41) | Polymerase | 581 - 600 bp |
| 6 | Ad41-R: TTTAGGAGCCAGGGAGTTATA |  |  |  |
| 7 | NV1a- F: ATGAATATGAATGAAGATGG | Norovirus  (NoV) | Polymerase | First round PCR |
| 8 | NV1b-F: ATGAACACAATAGARGATGG |  |  |  |
| 9 | NV7a-R: GGYCCYTCAGTYTTGTC |  |  |  |
| 10 | NV7-R: ATT GGT CCT TCT GTT TTG TC |  |  |  |
| 11 | NV6- F: TACCACTATGATGCAGATTA | Norovirus  (NoV) | Polymerase | 400 bp |
| 12 | NV6a- F: TATCACTATGATGCTGACTA |  |  |  |
| 13 | NV4-R: GTTGACACAATCTCATCATC |  |  |  |
| 14 | NV4a- R: ACAATYTCATCATCCCAT |  |  |  |
| 15 | NV4c-R: GTGCTGACGATCTCGTCATC |  |  |  |
| 16 | G1SK-F: CTGCCCGAATTYGTAAATGA | Norovirus  (NoV Genogroup I) | Capsid | 200 - 400 bp |
| 17 | G1SK-R: CCAACCCARCCATTRTACA |  |  |  |
| 18 | G2SK- F: CNTGGGAGGGCGATCGCAA | Norovirus  (NoV Genogroup II) | Capsid | 200 - 400 bp |
| 19 | G2SK- R: CCRCCNGCATRHCCRTTRTACAT |  |  |  |
| 20 | VP7 F: ATGTATGGTATTGAATATACCAC | Rotavirus A  (RVA-G) | Polymerase | 170 - 882 bp |
| 21 | VP7 R: AACTTGCCATTTTTTCC |  |  |  |
| 22 | VP4 F: TATGCTCCAGTNAATTGG | Rotavirus A  (RVA-P) | Polymerase | 130 - 664 bp |
| 23 | VP4 R : ATTGCATTTCTTTCCATAATG |  |  |  |
